# Using reporters of different misfolded proteins reveals differential strategies in processing protein aggregates

**DOI:** 10.1016/j.jbc.2022.102476

**Published:** 2022-09-09

**Authors:** Kara L. Schneider, Doryaneh Ahmadpour, Katharina S. Keuenhof, Anna Maria Eisele-Bürger, Lisa Larsson Berglund, Frederik Eisele, Roja Babazadeh, Johanna L. Höög, Thomas Nyström, Per O. Widlund

**Affiliations:** 1 Institute for Biomedicine, Sahlgrenska Academy, Centre for Ageing and Health – AgeCap, University of Gothenburg, Gothenburg, Sweden; 2 Department of Biology and Biological Engineering, Chalmers University of Technology, Gothenburg, Sweden; 3 Department for Chemistry and Molecular Biology, University of Gothenburg, Gothenburg, Sweden; 4 Department of Molecular Sciences, Uppsala BioCenter, Swedish University of Agricultural Sciences and Linnean Center for Plant Biology, Uppsala, Sweden

**Keywords:** protein aggregation, protein misfolding, proteostasis, *Saccharomyces cerevisiae*, yeast, spatial protein quality control, heat shock, chaperone, PQC, protein quality control, SIM, structured illumination microscopy, ts, temperature sensitive, tss, temperature-sensitive synthesis

## Abstract

The accumulation of misfolded proteins is a hallmark of aging and many neurodegenerative diseases, making it important to understand how the cellular machinery recognizes and processes such proteins. A key question in this respect is whether misfolded proteins are handled in a similar way regardless of their genetic origin. To approach this question, we compared how three different misfolded proteins, guk1-7, gus1-3, and pro3-1, are handled by the cell. We show that all three are nontoxic, even though highly overexpressed, highlighting their usefulness in analyzing the cellular response to misfolding in the absence of severe stress. We found significant differences between the aggregation and disaggregation behavior of the misfolded proteins. Specifically, gus1-3 formed some aggregates that did not efficiently recruit the protein disaggregase Hsp104 and did not colocalize with the other misfolded reporter proteins. Strikingly, while all three misfolded proteins generally coaggregated and colocalized to specific sites in the cell, disaggregation was notably different; the rate of aggregate clearance of pro3-1 was faster than that of the other misfolded proteins, and its clearance rate was not hindered when pro3-1 colocalized with a slowly resolved misfolded protein. Finally, we observed using super-resolution light microscopy as well as immunogold labeling EM in which both showed an even distribution of the different misfolded proteins within an inclusion, suggesting that misfolding characteristics and remodeling, rather than spatial compartmentalization, allows for differential clearance of these misfolding reporters residing in the same inclusion. Taken together, our results highlight how properties of misfolded proteins can significantly affect processing.

Maintenance of a functional proteome is crucial for longevity, reproductive capacity, and overall cellular fitness. An imbalance in protein homeostasis (proteostasis) is a hallmark of aging and linked to a number of age-related diseases (1, 2, 3, 4, 5). Proteostasis imbalance can result in aberrant proteins that disrupt cellular function by loss of function of the protein itself or by gain-of-toxic function, often associated with aberrant oligomers or aggregates that interfere with essential processes (6, 7, 8, 9). Therefore, cells rely on an intricate intracellular network of protein quality control (PQC) that maintains proteostasis and prevents proteotoxicity.

The PQC system consists of two interrelated and evolutionary conserved systems, the temporal and the spatial PQC (10, 11). A large network of chaperones, cochaperones, and nucleotide exchange factors constitutes the temporal PQC involved in proper folding of nascent polypeptides, refolding of aberrant proteins, and degradation of proteins *via* the ubiquitin-proteasome and vacuole/autophagy pathways. In parallel, the spatial PQC recognizes oligomers/aggregates and mitigates their toxicity by sequestering them into larger protein inclusions and depositing them at distinct protective sites (12, 13, 14, 15). While the characteristics of protein inclusions differ between organisms, the general phenomenon of sequestration of aberrant and misfolded proteins into inclusions is conserved from bacteria to humans (16, 17, 18, 19, 20). The yeast *Saccharomyces cerevisiae* has been a useful model in the study of PQC pathways in general and particularly suited for studies on spatial PQC and aging since protein aggregates have been shown to be subjected to a mother cell-biased asymmetric partitioning upon cell division leading to daughter cell rejuvenation (21, 22, 23, 24, 25).

To study the response of the spatial PQC pathways upon stress and aging, misfolded proteins need to be visualized and tracked in the cell. In *S. cerevisiae*, this typically involves the non-native expression of misfolding proteins tagged with a fluorophore to monitor aggregation and inclusion formation. A spectrum of reporters with different properties and genetic controls is available to test the responses of PQC pathways, as processing pathways can differ among reporters. In addition, investigating differences between reporters can reveal pathways active in specific cellular contexts or even uncover new pathways (10, 26, 27). In spite of differences between reporter proteins, most cytosolic reporter proteins follow a general spatial PQC pathway, in which they accumulate in small, visible aggregates mainly in the cytosol and at organelle surfaces (called stress foci/CytoQ/Q-bodies) upon proteostasis stress (12, 25, 28, 29) and eventually become sequestered into larger inclusions at distinct sites; the intranuclear/juxtanuclear quality control sites, the peripheral, vacuole-associated insoluble protein deposit (17, 28), and a site proximal to mitochondria (30, 31, 32, 33).

A variety of reporters exists for *S. cerevisiae*, however, these reporters can harbor disadvantages for investigating PQC. They are often nonyeast proteins, may require additional treatments or changes in carbon sources for induction of expression which changes the physiology of the cell, they do not provide suitable nonmisfolding controls, or are toxic for the cells due to secondary effects of proteopathy (reviewed in (27)).

Temperature-sensitive (ts) alleles are especially convenient to use as misfolding reporters since aggregation is rapidly induced by temperature shift and it allows investigation of the response to unfolding proteins at or near physiological conditions. In this work, we used different ts alleles, with their corresponding WT alleles as controls, to establish nontoxic direct reporters for misfolding and aggregation that allow us to detect possible variations in the processing of misfolding proteins upon heat shock. We utilized three ts alleles, *guk1-7*, *gus1-3*, and *pro3-1*, whose mainly cytosolic protein products have reported differences in degradation behavior (34, 35) and protein size. We report on the behavior of the reporters with a focus on spatial PQC upon a mild heat shock and with differences especially in their clearance during both continuous heat shock as well as recovery at permissive temperature. Strikingly, we see that different reporter proteins are cleared at differential rates even when they are intermixed within the same aggregate/inclusion.

## Results

### The misfolding reporters used do not affect cellular fitness

**Figure 1:**
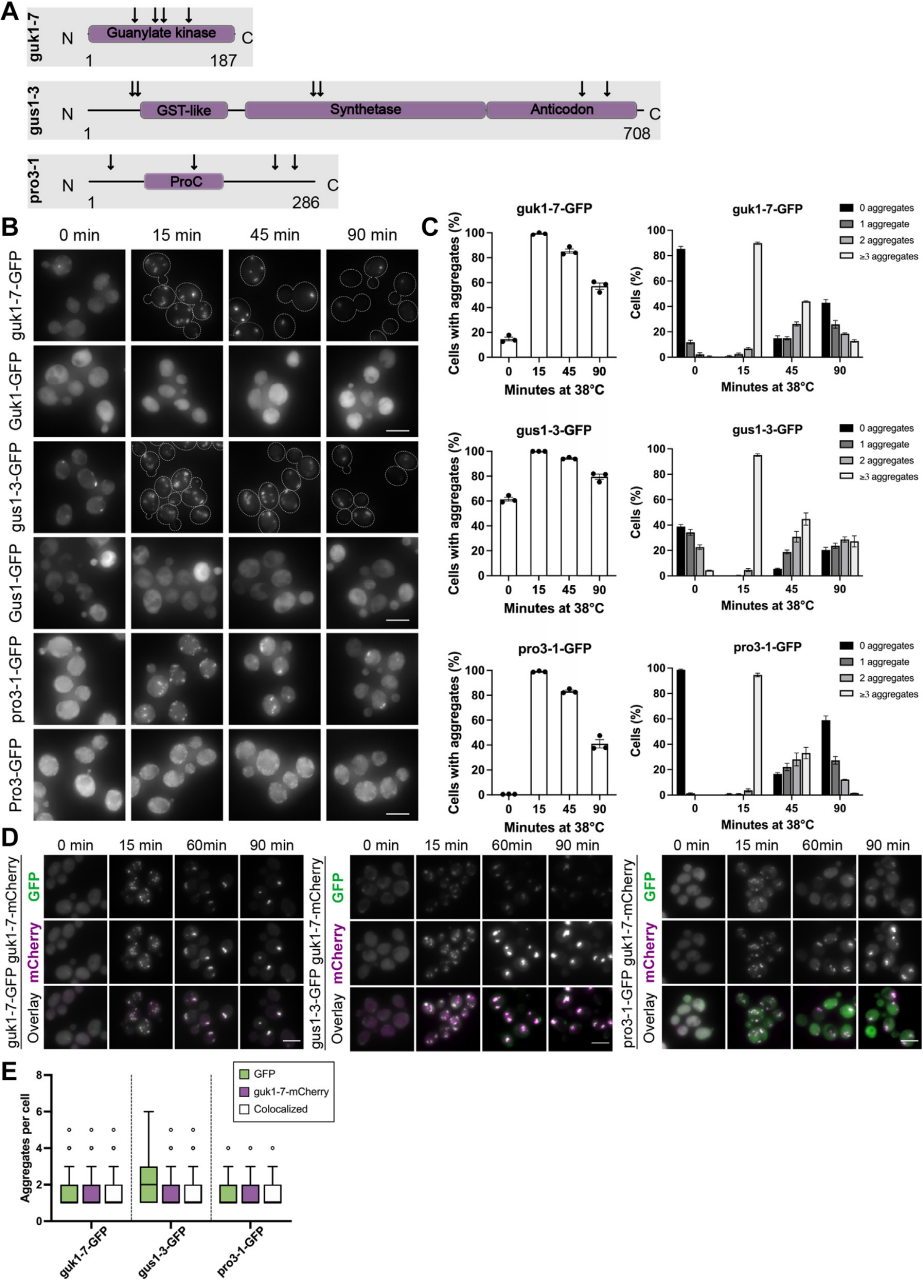
**The misfolding reporters aggregate during continuous heat shock and are subjected to common PQC pathways.***A*, domain representation of the three ts proteins guk1-7, gus1-3, and pro3-1 with locations of amino acid substitutions (*arrows*). Guk1 consists of 187 amino acids (aa) and the protein is mainly built of a guanylate kinase domain. guk1-7 contains the amino acid substitutions F58H, A84T, T95A, E127K. Gus1 consists of 708 aa and the amino acid changes in gus1-3 are F56L, F63L, M287I, D296G, I630T, K662E. It consists of a GST-like (70–170), a synthetase (205–509), and an anti-codon domain (509–699) (72). The domain organization of Pro3 is unclear. Based on homology modeling, the domains and mutations of Pro3 and pro3-1 have been presented previously (35); however, using BLAST, we identify a catalytic ProC superfamily domain from residues 70 to 170 (depicted in the figure). pro3-1 contains the amino acid substitutions A27T, V133A, H236L, K261E. *B*, time course of ts and WT alleles after shift from 30 °C to 38 °C (t = 0). *C*, quantification of inclusion formation and clearance (*left*) and aggregates per cell (*right*) of cells in (B). *D*, time course of strains containing a pairwise combination of reporters at 38 °C. Grayscale images of GFP and mCherry levels are not adjusted, whereas images labeled overlay are adjusted to better visualize colocalization. *E*, quantification of cells in (*D*) presented as a box plot (Tukey method). The scale bar represents 5 μm. PQC, protein quality control; ts, temperature sensitive.

The alleles of the three reporter genes are shown as domain representations in Figure 1*A*. Guk1 is a guanylate kinase that converts GMP to GDP (36, 37) and localizes to the cytoplasm and nucleus (38). Gus1 is a well-conserved glutamyl-tRNA synthetase that forms a complex with Arc1 and Mes1 and is found in the cytoplasm but also in the nucleus and mitochondria (39, 40, 41). Pro3 is a delta 1-pyrroline-5-carboxylate reductase involved in the proline biosynthesis pathway that is localized exclusively to the cytoplasm (42). We integrated fluorescently tagged versions of these alleles all under the control of a strong GPD promoter, into the *HIS3* locus, leaving the original WT loci intact (Fig. S1*A*). Thus, while the mutated reporters are ts, the strains are not with respect to growth.

To demonstrate their suitability as misfolding reporter proteins, we determined whether our overexpressed constructs have any detectable toxic properties. We first tested whether overexpression of the fluorescently tagged proteins themselves cause dominant negative effects. We did not observe any differences in growth rate between the WT control BY4741, the overexpressed WT alleles *GUK1-GFP*, *GUS1-GFP*, *PRO3-GFP*, and the overexpressed ts alleles (Fig. S1*B*). Additionally, fitness was not affected at higher temperatures, where the ts alleles are induced to misfold (37 °C and 39 °C) (Fig. S1*C*). Even when inducing global protein misfolding using the proline analog azetidine-2-carboxylic acid (43), overexpression of the misfolding reporters did not exhibit toxicity (Fig. S1*D*).

We next examined whether any toxicity can be seen during replicative aging. Protein aggregates arise in old cells in many organisms and are connected to a decline in both temporal and spatial PQC in old cells (11, 44, 45, 46, 47). Overloading the PQC continuously with expression of the misfolding reporters could therefore negatively affect lifespan. However, strains carrying the ts alleles and the corresponding WT alleles did not produce fewer daughters in comparison to BY4741 (Fig. S1*E*). We also tested whether these aggregates are asymmetrically inherited (23) and observed a similar degree of aggregate asymmetry among the reporters, which was comparable to the well-established aggregate marker Hsp104-GFP (Fig. S1*F*).

Taken together, the experiments show that these reporters are not toxic and have no noticeable effects on cellular functions, which makes them ideal to monitor PQC and makes them useful alternatives to the established ts model misfolding protein, Ubc9-ts, which can be severely toxic to the cell when continuously overexpressed (45).

### Misfolding reporters are mostly sequestered to common inclusion sites but differ in their distribution

The aggregation propensities and dynamics of the ts reporters were tested by subjecting them to continuous heat shock (38 °C). Aggregation was compared to that of nonheat shocked cells (30 °C) as well as to their corresponding WT reporters, Guk1-GFP, Gus1-GFP, and Pro3-GFP (Fig. 1*B*). During continuous heat shock, all three ts alleles formed a large number of aggregates at the initial time point (15 min) whereas the WT versions did not (Fig. 1, *B* and *C*). The numbers of aggregates diminished throughout the time course, indicating that aggregates are coalescing into larger inclusions and/or are disaggregated, degraded, or refolded. The pro3-1-GFP aggregates were most rapidly cleared out of cells and showed fewer inclusions per cell than the other misfolding proteins after 90 min (Fig. 1*C*). Overall, aggregates of guk1-7 and gus1-3 appeared more difficult to clear for the cell. Consistent with this observation, pro3-1-GFP was the only protein that showed an entirely diffuse and cytoplasmic signal at 30 °C with no evidence of aggregation, while guk1-7 and gus1-3 aggregated to a small degree, even at 30 °C.

We wondered whether the differences in protein aggregation arise because the misfolded proteins are sequestered into different compartments throughout heat shock. To approach this possibility, we monitored their colocalization in pairwise combinations of GFP- and mCherry-tagged variants of the misfolding reporters. After 90 min of heat shock, we observed mostly strong colocalization between all of them, showing that these misfolding proteins are mainly deposited at common PQC sites (Figs. 1*D* and S1*G*). In line with this, we also observed a high level of colocalization of the three reporters with the established misfolding reporter Ubc9-ts (Fig. S1*H*).

Interestingly, gus1-3 formed a fraction of “additional” aggregates, which did not colocalize with guk1-7 or pro3-1. This indicates that guk1-7 and pro3-1 are targeted by the same PQC machineries, while handling of gus1-3 likely involves additional pathways (Figs. 1*E* and S1*I*).

### The misfolded reporter proteins are cleared at differential rates and display differences in proteasome-dependent processing

**Figure 2:**
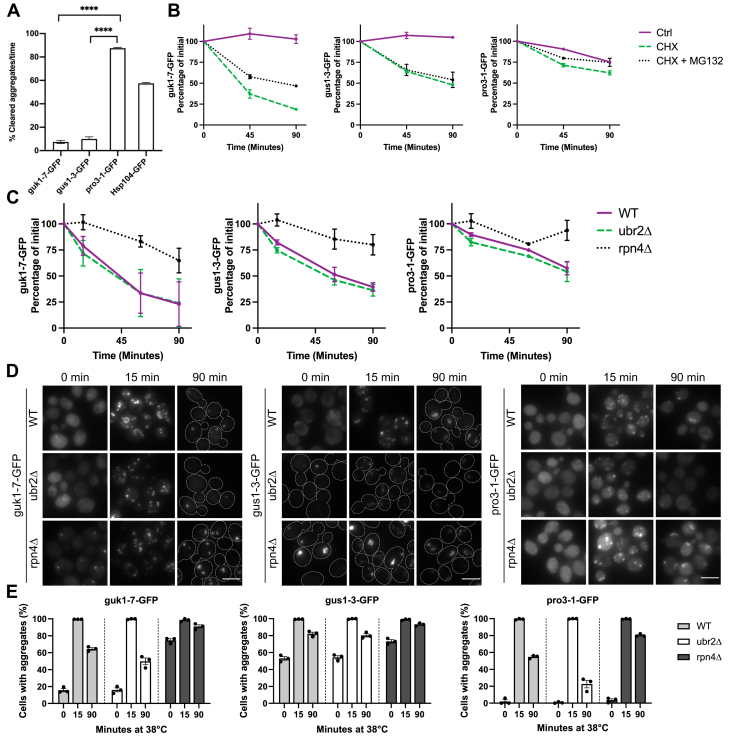
**pro3-1-GFP is cleared most efficiently and guk1-7-GFP and pro3-1-GFP are degraded by the proteasome but not gus1-3-GFP.***A*, pro3-1-GFP is cleared more successfully than guk1-7-GFP, gus1-3-GFP, and endogenous proteins visualized with Hsp104-GFP after heat shock at 38 °C (60 min) followed by recovery at 30 °C (60 min). Unpaired two-tailed *t* test. *B*, guk1-7-GFP and pro3-1-GFP but not gus1-3-GFP are stabilized by the proteasome inhibitor MG132 during heat shock at 38 °C with cycloheximide treatment. *C*, *ubr2Δ* cannot accelerate degradation of the reporter proteins but *rpn4Δ* decelerates degradation of all three reporter proteins when monitoring protein levels during heat shock at 38 °C with cycloheximide treatment. *D*, *ubr2Δ* increases inclusion clearance of guk1-7-GFP and pro3-1-GFP but not gus1-3-GFP during heat shock at 38 °C. *rpn4Δ* makes inclusion clearance less efficient for the three reporters. *E*, quantification of cells in (*D*). ∗∗∗∗*P* < .0001 The scale bar represents 5 μm.

Our observation that pro3-1 appeared to be handled most efficiently with respect to aggregate clearance (Fig. 1*C*) lead us to further investigate whether aggregates formed by guk1-7, gus1-3, and pro3-1 differ in their propensity to be disaggregated. We therefore determined their rates of clearance after induction of aggregation for 60 min followed by subsequent recovery at 30 °C. Strikingly, the aggregated reporter proteins were cleared with markedly different efficiencies. While aggregates of pro3-1-GFP were eliminated most rapidly (Fig. 2*A*), guk1-7-GFP and gus1-3-GFP were cleared much less efficiently, even at a slower rate than endogenous protein aggregates detected by Hsp104. The slower clearance rate of guk1-7 and gus1-3 most likely explains why so many aggregates of these misfolding reporters remain in the cell at later time points during continuous heat shock (Fig. 1*C*).

The variation in aggregate clearance of the misfolding reporters made us question whether they are differentially handled by the 26S proteasome, which is part of the temporal PQC responsible for protein degradation. We first observed that guk1-7-GFP is degraded most rapidly out of the three reporters when translation is shut off using cycloheximide (Fig. 2*B*). In accordance with a previous study (35), we see stabilization of guk1-7-GFP and pro3-1-GFP upon 26S proteasome inhibition with MG132 treatment, but not of gus1-3-GFP, indicating that proteasome activity is not mainly responsible for gus1-3-GFP turnover.

To complement chemical inhibition of the proteasome, we proceeded with genetic manipulations of transcriptional control factors of proteasome levels to investigate proteasomal degradation of the misfolding reporters (48). The transcription factor Rpn4 positively regulates expression of UPS-related target genes. Deletion of *RPN4* therefore reduces proteasome levels. In the absence of stress, the E3 ubiquitin ligase Ubr2 ubiquitylates Rpn4 for degradation. Depleting cells of Ubr2 thus leads to stabilization of Rpn4 and thereby higher proteasome levels and proteasome capacity (48). We verified these effects by monitoring Rpn5 (a proteasomal subunit component) levels (Fig. S2*A*). Using this genetic system for the misfolding reporters, we show that the increased proteasome capacity in the *ubr2Δ* mutant did not accelerate degradation of the misfolding reporters during heat shock (Fig. 2*C*). However, reduced proteasome capacity achieved by the *rpn4Δ* mutation stabilized all three misfolding reporters during heat shock, even gus1-3-GFP, which could not be stabilized by MG132. Stabilization of gus1-3-GFP was surprising and this prompted us to test whether gus1-3 as well as the other misfolding proteins were ubiquitylated. Indeed, all three misfolding reporter proteins were ubiquitylated, even the WT Gus1-GFP and Pro3-GFP constructs (Fig. S2*B*). We therefore conclude that all three misfolding proteins may be targets of the proteasome, albeit to different degrees. However, elevated proteasome capacity does not in itself improve degradation of the reporters used, suggesting a bottleneck in other processes required for proteasomal degradation, for example, ubiquitylation.

After confirming that the guk1-7 and pro3-1 proteins are degraded by the proteasome, we tested whether the genetic manipulations causing alterations in proteasome levels/capacity would affect aggregate levels during continuous heat shock with ongoing translation since the results may explain the differences observed in clearance and spatial handling of such aggregates. Mutations in *UBR2* and *RPN4* have previously been shown to affect aggregation of other reporters during different stresses (44, 48). An increase in proteasome levels caused by loss of *UBR2* was unable to decrease aggregation of guk1-7-GFP and gus1-3-GFP occurring at 30 °C (Fig. 2, *D* and *E*). Furthermore, gus1-3-GFP aggregation during heat shock was not influenced by the *ubr2Δ* mutation. In contrast, for guk1-7-GFP, and more so for pro3-1-GFP, we observed a faster clearance of aggregates during continuous heat shock until 90 min upon deleting *UBR2* even though the cells form aggregates at the early time point (15 min). This indicates that increased proteasome capacity aids in the aggregate clearance of the proteasome substrates guk1-7-GFP and pro3-1-GFP during continuous heat shock but not for gus1-3-GFP, which is in line with the results observed for turnover of the reporters upon MG132 treatment during heat shock (Fig. 2*B*). Loss of *RPN4*, on the other hand, affects the clearance of aggregates of all three reporters as we have previously seen when monitoring degradation kinetics of the corresponding proteins upon a cycloheximide chase (Fig. 2*C*). An *RPN4* deletion caused an increase in cells with guk1-7-GFP and gus1-3-GFP aggregates before heat shock, possibly due to the general overload of the UPS by endogenous proteins. pro3-1-GFP, in contrast, remained soluble in *rpn4Δ* cells (Fig. 2*E*).

### The reporter proteins require Hsp104 and Hsp70 for efficient sequestration into inclusions but Hsp104 recruitment efficiency differs between reporters

**Figure 3:**
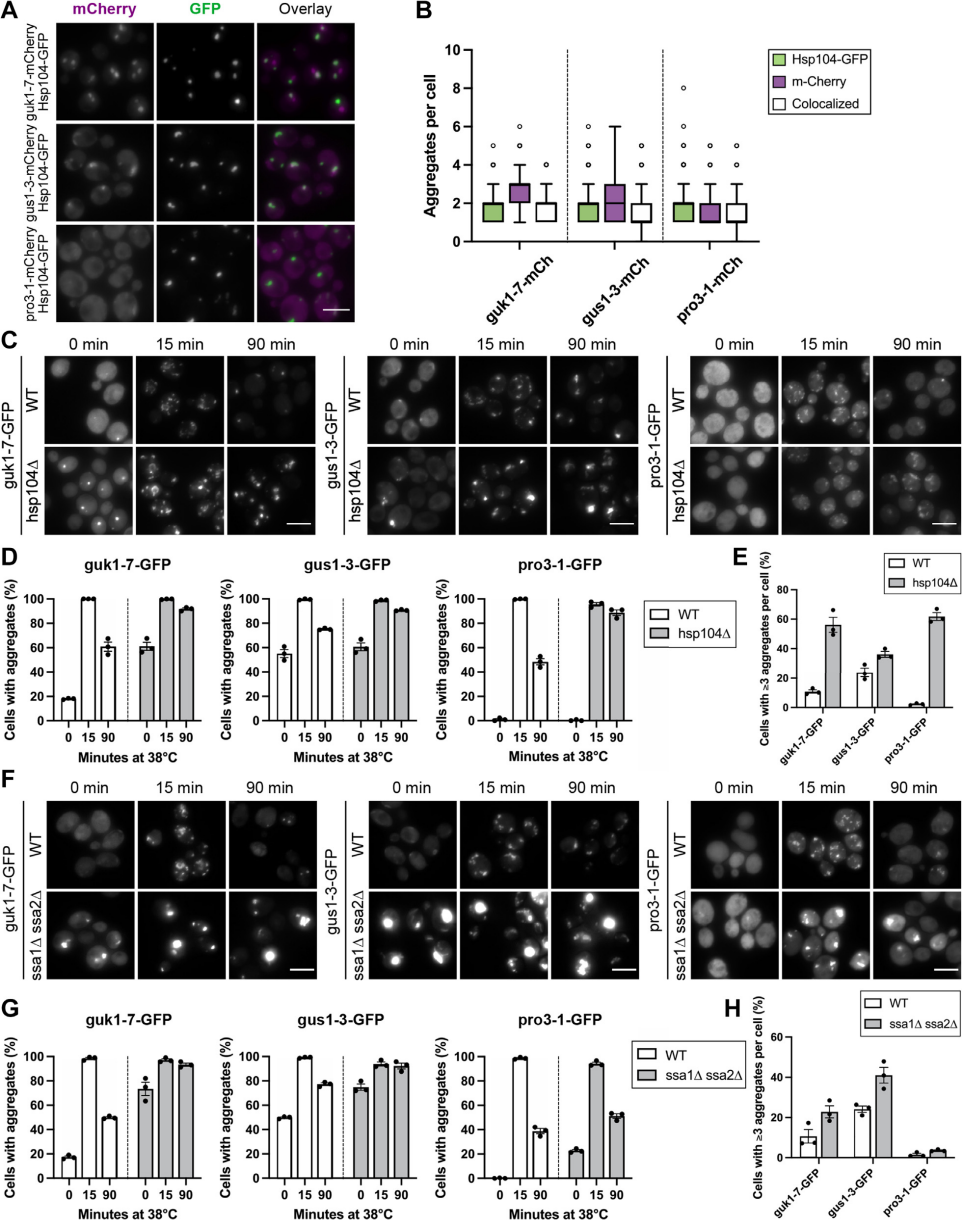
**Aggregates of the misfolding proteins recruit the disaggregase Hsp104 and their inclusion formation and clearance depend on Hsp104 and to some extent on Hsp70.***A*, colocalization between Hsp104-GFP and mCherry-tagged ts proteins after continuous heat shock at 38 °C for 90 min. *B*, quantification of cells in (*A*) presented as a box plot (Tukey method). *C*, inclusion formation of the ts proteins and their clearance during continuous heat shock at 38 °C are impaired in *hsp104Δ*. *D*, quantification of cells in (*C*). *E*, quantification of cells in (*C*) containing ≥3 aggregates per cell at 90 min. *F*, effect of *ssa1Δ ssa2Δ* on inclusion formation of the ts proteins and their clearance during continuous heat shock at 38 °C. *G*, quantification of cells in (*F*). *H*, quantification of cells in (*F*) containing ≥3 aggregates per cell at 90 min. The scale bar represents 5 μm. ts, temperature sensitive.

The general disaggregase, Hsp104, plays an important role in the clearance of protein aggregates. Since different misfolded proteins are cleared at different rates, we tested whether the aggregates formed by the misfolding reporters are differentially recognized by GFP-tagged Hsp104. All three reporter aggregates colocalized with Hsp104-GFP to a high extent upon continuous heat shock (Fig. 3*A*). However, guk1-7–containing cells mainly had three aggregates of guk1-7-mCherry, one of which lacked Hsp104-GFP (Fig. 3*B*). The “Hsp104-free” aggregate of guk1-7-mCherry was commonly found at the nucleus (91 ± 9% SD of non-colocalized guk1-7-mCherry aggregates were close to DAPI signal) (Fig. S3*A*), which might be related to Hsp104-GFP not being part of the juxtanuclear quality control compartment to the same extent as of the insoluble protein deposit (17) and to the endogenous cellular localization of WT Guk1 in the nucleus. For gus1-3, we observed the least overlap between Hsp104-GFP and gus1-3-mCherry aggregates but there was still colocalization of a median of one aggregate per cell. pro3-1-mCherry formed fewer aggregates than Hsp104-GFP but almost all aggregates formed by pro3-1 colocalized with Hsp104-GFP (92 ± 5% SD of pro3-1-mCherry aggregates colocalized with Hsp104-GFP) (Fig. S3*B*).

We next tested if Hsp104 is required for coalescence of the proteins into larger inclusions. Indeed, all three misfolding reporter proteins showed defects in inclusion clearance in an *hsp104Δ* background, which has previously been shown for other reporters (12, 25, 49) (Fig. 3, *C* and *D*) and failed to be efficiently sequestered into inclusions during a continuous heat shock (Fig. 3*E*). Thus, while Hsp104 is a common factor in the sequestration and disaggregation/removal of all three misfolding reporters, some misfolding proteins surprisingly form a subpopulation of aggregate species that do not significantly recruit Hsp104-GFP. The results also indicate that pro3-1 is most efficient in recruitment of Hsp104 to aggregates, which may facilitate the fast clearance of pro3-1 during heat shock. Likewise, the problematic clearance of gus1-3-GFP aggregates could be explained to some extent by the lack of recognition of a fraction of the aggregates by the disaggregase.

Hsp104 refolding activity requires the hsp70 homologs (50, 51). We therefore wanted to analyze the effect of impairing Hsp70 function on aggregate formation and clearance. There are four cytosolic Hsp70s in yeast, Ssa1-4. The four isoforms are nearly identical and presence of a single Ssa is sufficient to ensure cellular growth (52). However, the functional redundancy of the Ssas is limited with regards to several PQC pathways (53). Deleting both *SSA1* and *SSA2* causes increased heat sensitivity and defects in spatial PQC, such as impaired Hsp104 recruitment to aggregates and their clearance. We thus used the *ssa1Δ ssa2Δ* background to monitor the behavior of the misfolding proteins upon compromised Hsp70 function (Fig. 3*F*). As previously reported, guk1-7-GFP and gus1-3-GFP have more aggregates in *ssa1Δ ssa2Δ* than the WT at 30 °C (53). We observed the same for pro3-1-GFP (Fig. 3*G*). Upon heat shock, almost all cells form visible aggregates in both *ssa1Δ ssa2Δ* and in the WT. The Hsp70 mutant cells showed substantial inclusions with high fluorescence intensity and diminished aggregate clearance during the time course, while we observed no major defect in aggregate coalescence (Fig. 3*H*).

### *pro3-1-GFP* is a ts synthesis allele

**Figure 4:**
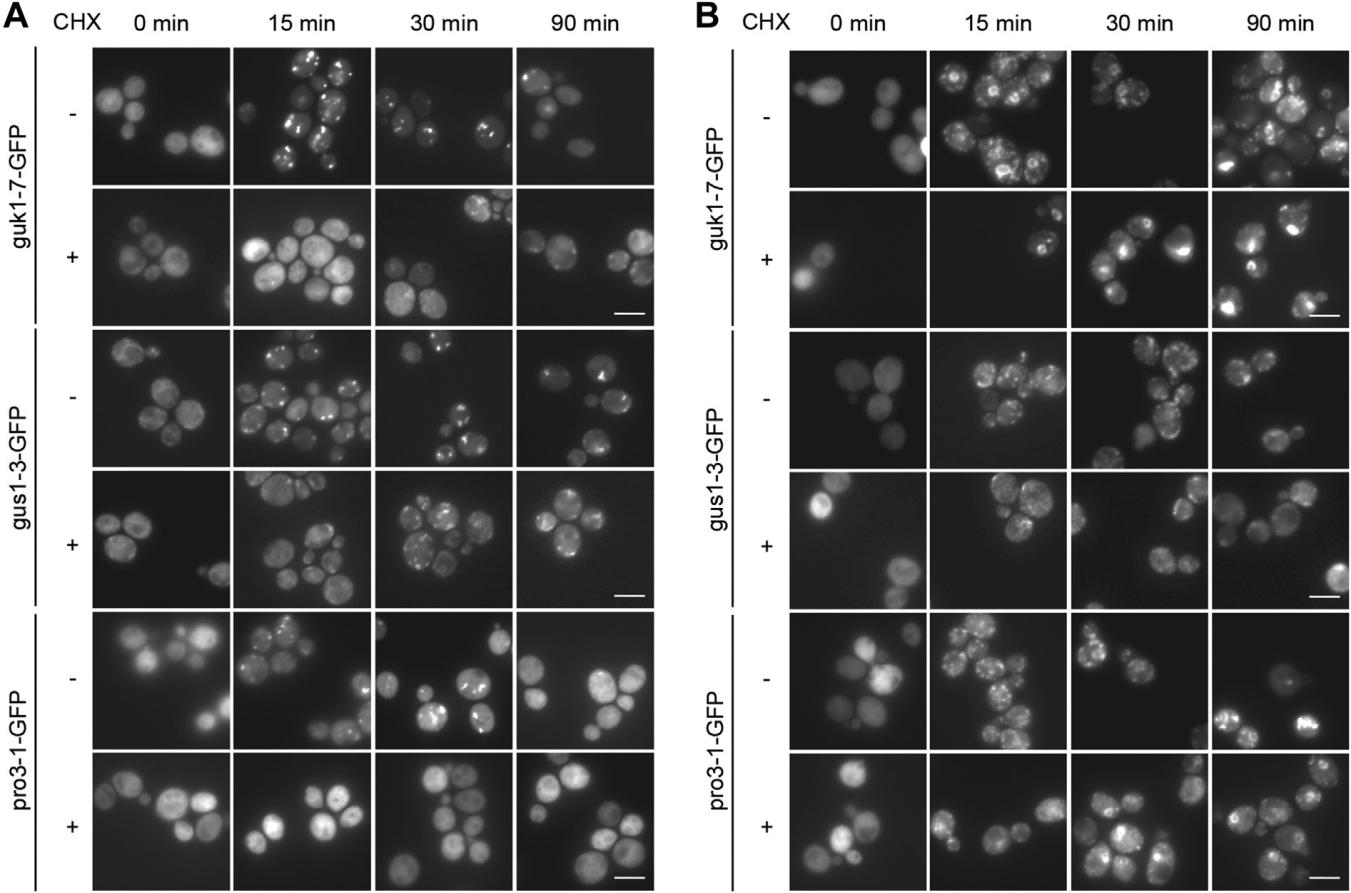
**pro3-1-GFP is a temperature-sensitive synthesis allele, while guk1-7-GFP and gus1-3-GFP are thermolabile.***A*, guk1-7-GFP and gus1-3-GFP form aggregates upon heat shock at 38 °C with (+) and without (−) translation inhibition, while pro3-1-GFP does not form aggregates at 38 °C upon cycloheximide treatment. *B*, at 42 °C, all three reporter proteins misfold independently of translation inhibition. Note circular accumulations of misfolding protein. Images in single Z-plane, intensity levels adjusted for optimal visualization. The scale bar represents 5 μm.

We noticed that pro3-1 formed inclusions to a lower extent directly after heat shock compared to guk1-7 and gus1-3 as there was a significant signal in the cytoplasm. This signal is likely pro3-1 protein that is either not misfolding or has rapidly been refolded. We therefore tested whether pro3-1 could be a temperature-sensitive synthesis (tss) allele (54, 55). Tss proteins are characterized by misfolding only during active translation, that is, misfolding as they emerge at the ribosome as nascent polypeptides. For this, we monitored aggregation using fluorescence microscopy with and without addition of cycloheximide to inhibit translation. Indeed, we found that pro3-1 remained soluble when translation was blocked (Fig. 4*A*). However, when we increased the temperature of heat shock to 42 °C, all three misfolding reporters formed aggregates independent of translation, suggesting that previously synthesized and folded pro3-1 will eventually misfold above a critical temperature (Fig. 4*B*).

We also observed distinct structures formed upon heat shock at the higher temperature for guk1-7 and pro3-1, including ring-shaped assemblies of fluorescently tagged protein, which correspond to a nucleolar structure that has been observed previously (45, 53) (Fig. S4). They are also visible, albeit less pronounced, at early time points (15 min) of heat shock at 38 °C.

### The individual misfolding reporter aggregates are cleared at different rates in common inclusions

**Figure 5:**
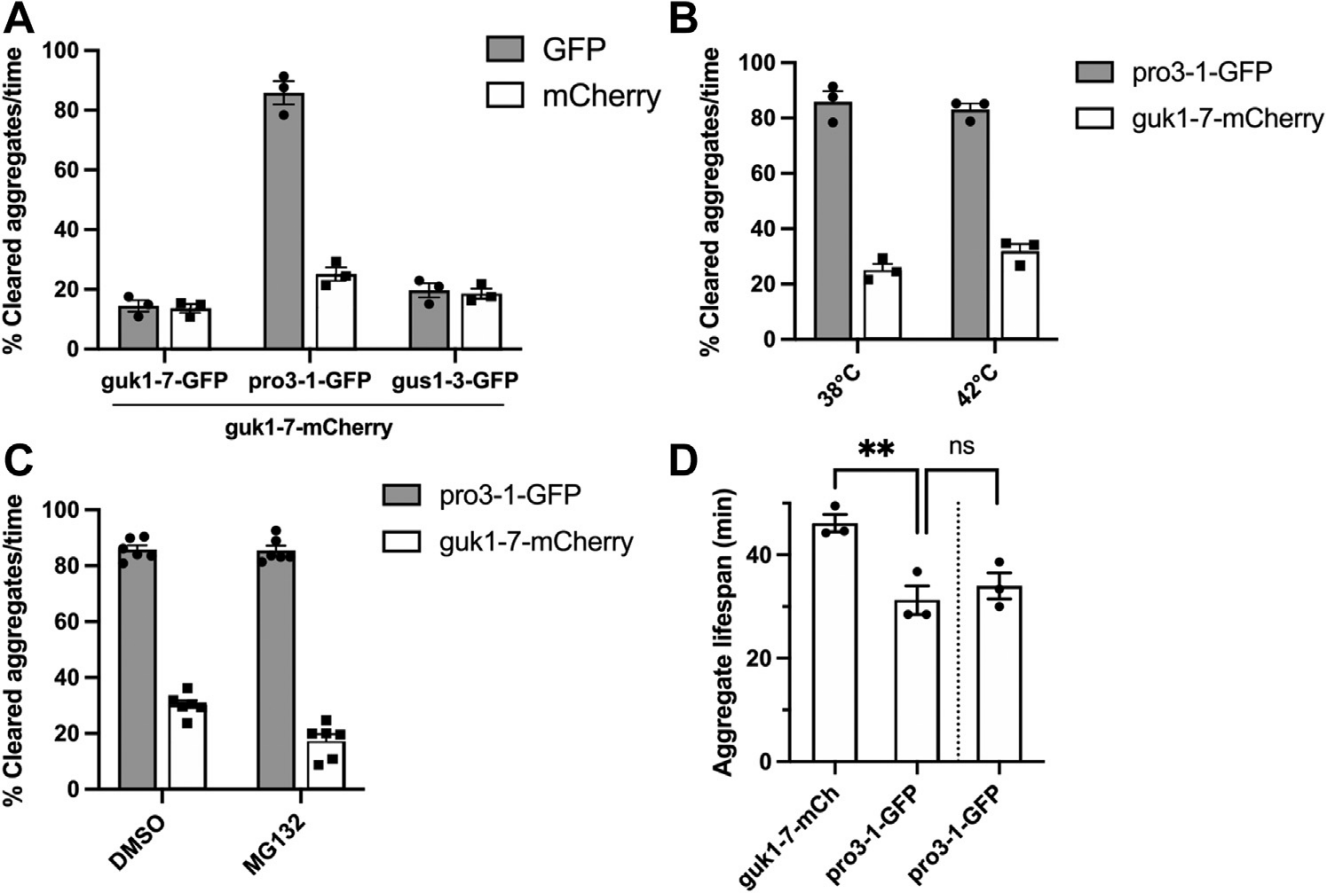
**pro3-1-GFP clearance is not affected in combination with guk1-7-mCherry.***A*, guk1-7-mCherry was combined with the three GFP-tagged ts alleles, and clearance of aggregates over time was monitored after heat shock at 38 °C for 60 min and recovery at 30 °C for 60 min. *B*, clearance of pro3-1-GFP aggregates in combination with guk1-7-mCherry is not decelerated when inducing aggregation at 42 °C. *C*, clearance of pro3-1-GFP aggregates in combination with guk1-7-mCherry is not decelerated upon proteasome inhibition. *D*, aggregate lifespan of mixed inclusions of guk1-7-mCherry and pro3-1-GFP and of a strain containing only pro3-1-GFP. Measurements were performed with timelapse microscopy of both strains mixed on an agar pad to monitor disaggregation at 30 °C after heat shock at 38 °C for 60 min. Images were taken every 7.5 min and aggregate lifespan was determined as the last time frame in which an aggregate was visible. ts, temperature sensitive.

All three reporters displayed a high level of colocalization among one another indicating that they are part of the same protein aggregate. We therefore suspected that the presence of misfolding protein that is not easily cleared (*i.e.*, gus1-3 or guk1-7) could retard clearance of one that is (such as pro3-1) when present in the same inclusion. Surprisingly, when we monitored the clearance of the individual reporters in strains with pairwise combinations, their clearance efficiencies were unaffected by the presence of another misfolding protein in the same inclusion (Figs. 5*A* and S5, *A*–*C*). pro3-1 was cleared out of cells as efficiently in combination with guk1-7 and with gus1-3 as by itself (Fig. 2*A*). The result of pro3-1 in combination with guk1-7 is especially interesting since almost all pro3-1 aggregates recruit Hsp104 and almost all pro3-1 aggregates colocalize with guk1-7 aggregates. Consequently, there are aggregates in the cell that contain both pro3-1 and guk1-7 and are recognized by Hsp104, yet pro3-1 continues to be removed from these aggregates faster than guk1-7. This difference was not due to the tss nature of pro3-1 as this difference in clearance was also seen in aggregates formed at 42 °C, a temperature at which pro3-1 also misfolds when translation is blocked (Fig. 5*B*). The faster clearance of pro3-1 out of protein inclusions is also not delayed by proteasome inhibition (Fig. 5*C*).

To find the difference in clearance on an aggregate level, we performed timelapse experiments and found that aggregate lifespan of pro3-1-GFP is significantly shorter than guk1-7-mCherry (Fig. 5*D*). Strikingly, this is unaffected by being combined with guk1-7-mCherry within the same inclusions. Thus, when present in the same aggregate as visualized by wide-field light microscopy, pro3-1-GFP is removed faster than guk1-7-mCherry and is in no way hindered in its clearance by presence of guk1-7-mCherry within the same aggregate. This process is facilitated by an Hsp104-dependent PQC pathway since both aggregates remained colocalized and uncleared during a disaggregation timelapse experiment in *hsp104Δ* cells (Fig. S5*D*).

### Structured illumination microscopy and EM of protein aggregates of pairwise-combined reporters indicate no spatial separation of differentially tagged misfolding proteins within a shared inclusion

**Figure 6:**
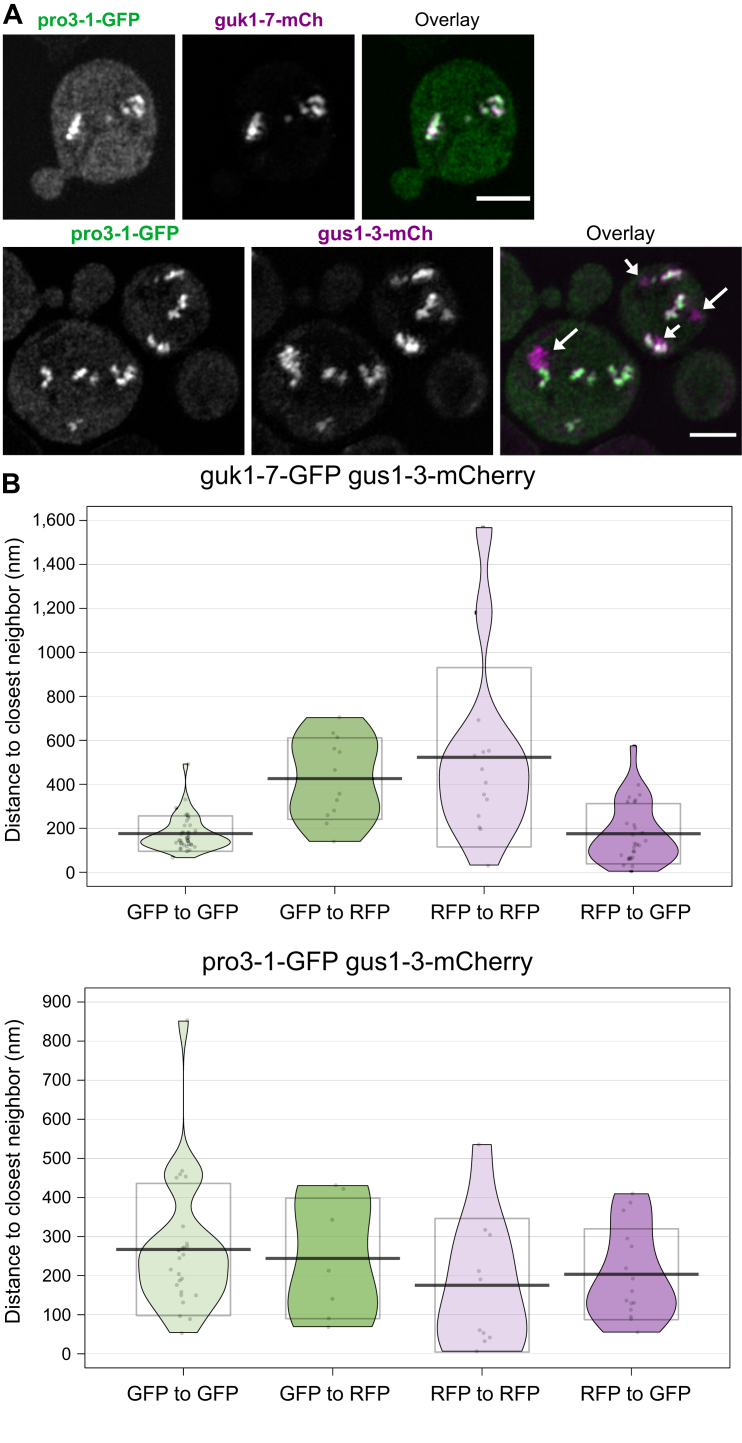
**The different protein species are mixed within common inclusions.***A*, 3D-SIM images of aggregates of pro3-1-GFP guk1-7-mCherry and of pro3-1-GFP gus1-3-mCherry after 60 min at 38 °C shown as maximum Z-projections. *Arrows* indicate gus1-3-mCherry aggregates that do not colocalize with pro3-1-GFP. The scale bar represents 2 μm. *B*, nearest-neighbor analysis of gold beads attached to anti-GFP (15 nm) or anti-RFP (25 nm) within aggregates (38 °C, 30 min) of cells containing guk1-7-GFP gus1-3-mCherry and pro3-1-GFP gus1-3-mCherry visualized with EM. The plots labeled “GFP to GFP” contain the Euclidian distances scored between 15-nm beads, “GFP to RFP” contain the distances between 15-nm and 25-nm beads, “RFP to RFP” contain the distances between 25-nm beads, and “RFP to GFP” contain the distances between 25-nm and 15-nm beads. Graphs show the mean (*line*) and SD.

Our observation that one misfolded protein species could be cleared faster from an aggregate consisting of two different species was somewhat surprising. We therefore considered the possibility of an intra-aggregate compartmentalization that allows for better accessibility of pro3-1 to PQC factors that was not discernable by conventional wide-field fluorescence microscopy. However, when resolution was increased using 3D super resolution microscopy (3D-structured illumination microscopy (SIM), Fig. 6*A*), the inclusions of combined protein species of both the control strain guk1-7-GFP guk1-7-mCherry and the strain of interest pro3-1-GFP guk1-7-mCherry appeared to consist of evenly mixed fluorescent protein. The individual channels show that the reporter aggregates have the same shape, resulting in a clear overlay. Similarly, the control strain gus1-3-GFP gus1-3-mCherry (and guk1-7-GFP guk1-7-mCherry) showed colocalization (Fig. S6*A*) but the previously described “additional” aggregates of gus1-3-mCherry became visible in combination with pro3-1-GFP (Fig. 6*A*). Consequently, 3D-SIM confirmed our previous observations from conventional fluorescence microscopy.

We increased resolution further by performing EM of high pressure-frozen cells. Double-immunogold labeling allowed detection of the respective protein species within the inclusion, which are defined as an electron-dense ribosome-excluding area in the cytosol. To discern the different protein species, the secondary antibody recognizing RFP (gus1-3-mCherry) contained 25-nm gold beads and the one recognizing GFP (guk1-7-GFP or pro3-1-GFP) contained 15-nm gold beads (Fig. S6*B*). Nearest-neighbor analysis indicated that for both guk1-7-GFP gus1-3-mCherry and for pro3-1-GFP gus1-3-mCherry, there was no spatial separation between the different protein species within the inclusion as the mean distances within GFP-labeling gold beads did not significantly differ from the mean distances of RFP- to GFP-labeling gold beads (Fig. 6*B*). Accordingly, the distances did not differ significantly in the reverse analysis of within RFP beads and RFP-GFP beads.

## Discussion

In this study, we establish reporter constructs based on ts alleles from Khosrow-Khavar *et al.* to study PQC machineries with a focus on spatial PQC. The misfolding reporters have several advantages for the purposes of our study compared to other reporter systems. They are constitutively overexpressed and thus do not require any induction of expression that constitute a shift in itself (*e.g.*, galactose induction). They are integrated into the genome, which leads to relatively equal expression levels throughout the cell population and allows performance of experiments in rich media. Importantly, the overproduction of the proteins does not show toxicity in our assays and the WT alleles *GUK1*, *GUS1*, and *PRO3* remain unaffected as the misfolding reporters are integrated in the *HIS3* locus. Use of mutated versions of native yeast proteins also means that the proteins of the WT alleles can be used as folding controls, which are often unavailable for other model proteins. Another advantage of these reporter constructs is that they facilitate monitoring of PQC directly by tagging a misfolding protein, instead of following pathways indirectly with Hsp104-GFP.

We believe the misfolding reporters will prove useful in answering questions in the field of spatial PQC, which are not fully resolved. For example, proteins generally harbor the potential to misfold but only a small fraction of them has been linked to proteopathies; consequently, toxic and nontoxic misfolding proteins may have characteristics causing them to be handled in different ways by the PQC systems. The nontoxic yeast reporters presented here will allow us to investigate and distinguish fundamental mechanisms of aggregation and quality control in combination with disease model proteins. Furthermore, while the misfolding reporter proteins do not affect replicative lifespan, they will be useful to study aging nonetheless. Open questions are which PQC pathways handle these reporters during aging and if their spatial PQC is compromised at some point during the replicative lifespan of cells as it has been described for endogenous protein aggregates visualized with Hsp104-GFP and for other reporters (10, 45, 47, 56, 57). The constructs will allow investigation of directly tagged misfolding protein during aging at close to native conditions.

We used these constructs to elucidate what, if any, differences exist in the processing of similarly expressed model misfolding proteins, guk1-7, pro3-1, and gus1-3. The data herein demonstrate common PQC pathways especially for guk1-7-GFP and pro3-1-GFP. Both reporters are handled more successfully by temporal and spatial PQC than gus1-3-GFP, which might be due to them being more efficiently targeted to the proteasome. Of the three proteins studied here, gus1-3 poses the biggest challenge for the cell. This might be due to its large size, its presence in a complex, or its partial localization to mitochondria. Of special interest for future investigations is the fraction of unusual aggregates formed by gus1-3, which do not colocalize with the other reporter proteins and are not recognized by Hsp104.

Clearance assay shows that misfolded pro3-1-GFP is rapidly processed compared to guk1-7 and gus1-3. One potential reason for this is more efficient general recruitment of Hsp104. Additionally, pro3-1 appears to be stable at 30 °C and only misfolds during active translation. It is therefore considered less prone to misfolding and may also be more efficiently refolded as a consequence.

Normal proteasome function is required for efficient removal of the three substrates studied here as they were all stabilized by deletion of *RPN4*. However, in agreement with previous work (35), proteasome inhibition using MG132 failed to stabilize gus1-3, indicating striking differences in how these three misfolding proteins are handled by the proteasome. Furthermore, a boost of proteasome activity using *ubr2Δ* did not facilitate faster degradation during translation inhibition (35). It did, however, result in more efficient clearance of guk1-7 and pro3-1 aggregates during continuous heat shock. Hence, there may be a translation-dependent mechanism required during heat shock that aids in proteasomal clearance of guk1-7-GFP and pro3-1-GFP in *ubr2Δ*, which is masked due to cycloheximide treatment in the degradation assays but is visible during continuous heat shock. One possibility is that ubiquitin forms a bottleneck for guk1-7 and pro3-1 degradation upon heat shock with concomitant cycloheximide treatment, as ubiquitin is known to become depleted during translation inhibition. Together with global protein stress upon heat shock, ubiquitin levels may be insufficient for *ubr2Δ* to enhance proteasomal degradation (58). Another possible explanation is that the beneficial effect of *ubr2Δ* on degradation is not visible in our assay because addition of cycloheximide hinders not only the production of the misfolding reporters but also of proteins required for adequate heat shock response upon shift to 38 °C, thereby leading to a lack of factors that aid the proteasome in degradation of the misfolding proteins.

*Hsp104Δ* and *ssa1Δ ssa2Δ* cells are deficient in controlling aggregate formation (30 °C), sequestration into inclusions (cells ≥3 aggregates at 90 min, 38 °C), and aggregate clearance (cells with aggregates at 90 min, 38 °C) of all three reporters to various degrees. Overall, the defects in PQC in the Hsp100 and Hsp70 mutant strains we observed here are in line with previous reports for other systems to monitor PQC. Interestingly, the results are not entirely congruent even though Hsp70 and Hsp100 form a bi-chaperone system. Compromising *SSA1* and *SSA2* did not have severe effects on inclusion formation for the three reporters, in stark contrast to cells lacking Hsp104. Since the *ssa1Δ ssa2Δ* cells showed clearance defects even though they were mostly able to sequester aggregates into inclusions, there may be additional pathways relevant for their clearance. Of note is that the Hsp70 mutant showed an accumulation of pro3-1-GFP aggregates at standard growth temperature, even though this misfolding reporter remained stable in other proteostasis mutants such as *rpn4Δ*. It is possible that *SSA1/SSA2* are required in a specific PQC pathway that ensures proper pro3-1 folding or its degradation/refolding. Alternatively, the *ssa1Δ ssa2Δ* mutant may overwhelm an Hsp70-independent system that generally handles pro3-1-GFP but is overloaded due to the large pro3-1-GFP accumulation (high fluorescence intensity of the reporters in *ssa1Δ ssa2Δ*).

We exploited the variation in the processing we observed with the reporter proteins to investigate pairwise combinations of misfolding proteins with different clearance dynamics. We show that in spite of deposition of aggregates into common compartments, they do not influence each other’s disaggregation efficiencies. Based on the imaging performed with SIM and EM, the protein species are intermixed within common aggregates and there is no large inter-aggregate organization that compartmentalizes the different reporters. Instead, it is likely that the faster clearance of pro3-1-GFP is related to its relatively high stability at 30 °C. When shifting the cells to 30 °C for recovery after heat shock, pro3-1-GFP may be able to refold and stabilize, in contrast to guk1-7-mCherry, which is misfolded at 30 °C to some extent. Additionally, any newly synthesized protein of pro3-1-GFP can be properly folded during the recovery phase, while guk1-7-mCherry partially misfolds and may be fed to already existing aggregates, thus displaying a slower aggregate clearance dynamic. Importantly, the clearance of pro3-1-GFP is not slowed down by the presence of guk1-7-mCherry in the same aggregate. This indicates that the protein species are targeted by PQC machineries independently of one another allowing for differential recruitment of, for example, disaggregases. Alternatively, the aggregate may be remodeled by the disaggregation machinery to allow continued access to the more easily refolded/degraded substrate. In agreement with both options, faster clearance of pro3-1-GFP in mixed aggregates is facilitated by an Hsp104-dependent pathway.

## Experimental procedures

### Yeast media, plasmids, and strains

*S. cerevisiae* strains and genotypes used in this study are listed in Table S1. Deletion mutants *hsp104Δ*, *ubr2Δ*, and *rpn4Δ* stem from the yeast knockout library (BY4741 background). Deletion of *HSP104* in the pro3-1-GFP guk1-7-mCherry strain was achieved by PCR-mediated knockout using pFA6a–hphNT1 (59). The guk1-7, gus1-3, and pro3-1 plasmids were constructed based on plasmids from the Mayor lab. BPM458 (guk1-7GFP), BPM500 (gus1-3GFP), and BMP507 (pro3-1GFP) (Mayor lab) were cut with ApaI/SacI and ligated into ApaI/SacI linearized pRS403 to create the GFP-tagged pPW349, pPW350, and pPW351 integrating into *HIS3*. The construction of the mCherry-tagged plasmids was performed by cutting pPW350 with SacI/ApaI to move gus1-3-GFP into pRS405. Bases 735 to 1315 of *LYS2* were amplified and inserted using XhoI/ApaI for integration in the yeast genome. mCherry was amplified from pBS35 (yeast resource center) and inserted using XbaI/AscI to create pPW399. guk1-7 was moved into pPW399 from pPW350 using SacI/XbaI to create pPW413. pro3-1 was cloned in using SacI/NotI to create pPW418. All experiments were conducted in rich media, YPD with 2% glucose. Strains containing pSik1-RFP or pUbc9s-GFP were cultured in synthetic drop out media.

### Aggregation time course and aggregate clearance

For aggregation time course analysis, cells were grown at 30 °C until mid-exponential phase (A_600_∼0.5). The initial time point 0 prior to heat shock was sampled and the cells were shifted to 38 °C for continuous heat shock. Cycloheximide was added to a final concentration of 250 μg/ml where indicated. Samples were taken at the indicated time points, fixed in 3.7% formaldehyde (final concentration), washed with PBS, and imaged using a conventional fluorescence microscope, the Zeiss Axio Observer.Z1 inverted microscope equipped with an Axiocam 506m camera and a Plan-Apochromat 100×/1.4 NA Oil DIC M27 objective. Images were taken with Z-stacks except where indicated and manually quantified using the CellCounter Plugin in Fiji (ImageJ) and number of aggregates per cell was determined. This data was used to calculate cells with aggregates (%) and also pooled into categories to determine fraction of cells containing zero, one, two, or ≥three aggregates per cell. Representative images of microscopy experiments are shown in maximum Z-projection of relevant Z-steps except where indicated. Fluorescence intensity levels are not adjusted during continuous heat shock except where indicated.

For microscopy of nucleolar deposits, cells were grown at 30 °C until mid-exponential phase (A_600_∼0.5), shifted to 42 °C for 30 min, and subjected to DAPI staining as described previously (53). Live cells were imaged directly.

For microscopy of Ubc9ts-GFP aggregates, cells were precultured in -Ura wih 2% raffinse and 0.5% glucose. Main cultures were grown to A_600_∼0.5 in 2% galactose to induce pUbc9ts-GFP expression and shifted to 38 °C at which time expression was shut off by addition of 2% glucose. Cells were imaged at indicated time points.

For aggregate clearance measurements, cells were grown at 30 °C until mid-exponential phase (A_600_∼0.5). The cells were shifted to 38 °C (or 42 °C where indicated) for 60 min (or 30 min where indicated) to induce protein aggregation, followed by recovery for 60 min (or 30 min where indicated) at 30 °C. Cells were fixed in 3.7% formaldehyde (final concentration) and treated as described for aggregation time course analysis. Images were quantified by ImageJ software (https://imagej.nih.gov) and fraction of cells (%) which could completely clear out their aggregates (cells without aggregates) was determined. For aggregate clearance with MG132 treatment, MG132 (Enzo Life Sciences, BML-PI102-0005, 5 mg) dissolved in DMSO (dimethyl sulfoxide) to the stock concentration of 75 mM was added to the cells after heat shock (60 min, 38 °C) at the final concentration of 100 μM supplemented with 0.003% SDS. The control experiment was supplemented with DMSO and 0.003% SDS in parallel. The aggregate clearance assay was performed as described.

### Timelapse microscopy

Timelapse microscopy was performed as described previously with minor modifications (60). Cells were grown to mid-exponential phase (A_600_∼0.5) at 30 °C in YPD. The cells were shifted to 38 °C for 60 min to induce aggregation. Cells were gently spun down and placed onto a 2% agar pad perfused with yeast nitrogen base) supplemented with complete supplement mixture) and 2% glucose (w/v) and sealed with a coverslip as described previously (61). The sample was imaged in 7 × 0.5 μm Z-steps for at least 60 min at 7.5 min intervals for aggregate lifespans and for 165 min for *hsp104Δ* (Fig. S5*D*, shown until time point 120 min). Temperature was maintained at 30 °C to monitor aggregate clearance using the TempModule S1 (Zeiss), Y-module S1 (Zeiss), Temperable insert S1 (Zeiss), Temperable objective ring S1 (Zeiss), and Incubator S1 230V (Zeiss). Focus was achieved using Definite Focus. All images were acquired using a Zeiss Axio Observer.Z1 inverted microscope with Axiocam 506 camera (Zeiss) with a Plan-Apochromat 100×/1.40 Oil DIC M27 objective (Zeiss), equipped with the standard filter sets (Zeiss): 38 HE Green Fluorescent Protein, 45 Texas Red, 49 DAPI. Timelapse images were processed with Fiji software (https://fiji.sc/) using the manual drift correction plugin. For representation, enhancements in brightness/contrast were used.

### Structured illumination microscopy

Mid-log cells heat shocked at 38 °C for 60 min were fixed, washed in PBS, and placed between microscope slide and coverslip for imaging with a ELYRA PS.1 system (Zeiss), an inverted microscope equipped with a Plan-Apochromat 63×/1.4 oil immersion objective. The filter modules used to detect GFP and mCherry were 488 EF BP 495-550/LP 750 SR and 561 EF BP 570-620/LP 750 SR. The lasers were solid-state 488 nm (100 mW, set to 2.2–2.8% depending on the misfolding protein) and solid-state 561 nm (100 mW, 2.1–2.5%). Exposure time for acquisition depended on the misfolding protein (85–100 ms). The microscope is equipped with an Andor iXon 885 EMCCD camera for SIM and gain was set to 60. Settings for SIM were 0.34 μm grating period, five rotations. Images of aggregates were acquired in 20 to 25 Z-steps depending on aggregate size at 0.11 μm each. After acquisition, SIM images were generated from raw data with the Structural Illumination function in Zen Black software (Zeiss, version 14.0.12.201). Due to the different filters used for acquiring GFP and mCherry channels, there was a small 3D displacement between the imaged volumes. To compensate for this, channel alignment was performed with the transform function in Fiji by adding a control strain for inclusions composed of one protein species (gus1-3-GFP-mCherry). Specifically, cells of interest were mixed on the coverslip with control cells containing the tandem-tagged misfolding protein (gus1-3-GFP-mCherry) and an additional tag that allows distinction from the cells of interest (Ina1-GFP, localizes to plasma membrane). Image transformation was achieved by manually aligning aggregates in both channels for gus1-3-GFP-mCherry Ina1-GFP–containing cells, which were in close proximity to cells of interest in the same field of view. For visualization, only the cells with reporters of interest are shown.

### Sample preparation, immunogold labeling, and EM

Cells were grown to A_600_∼0.5 at 30 °C in YPD. The cells were shifted to 38 °C for 30 min to induce protein aggregation. Cells were separated from the culture medium using a 0.22 μm filter and scraped off the filter membrane and high-pressure frozen in an Aluminum sample carrier using a Wohlwend Compact 3 (62, 63, 64, 65). The samples underwent freeze substitution in a Leica AFS2 and were incubated in 2% uranyl acetate (SPI supplies) diluted in nine parts acetone and one part methanol for 1 h (66), rinsed in acetone, and step-wise embedded in Lowicryl HM20 resin (Polysciences) at −50 °C, followed by a 5 days polymerization under UV light while allowing the samples to reach room temperature. Polymerized resin blocks were sectioned to 70 nm using a Reichert Ultracut S and placed on Formvar-coated 200-mesh copper grids.

Immuno labeling was performed using 1/10 and 1/30 dilutions of mouse anti-RFP (6G6, Chromotek) and rabbit anti-GFP (ab6556, Abcam) primary antibodies in block buffer, respectively. Primary antibodies were incubated overnight at 4 °C after fixation in 1% paraformaldehyde for 10 min and blocking in 0.8% bovine serum albumin in PBS with 0.1% fish skin gelatin for 1 h. The secondary antibodies, 25 nm (F’Ab) goat anti-mouse (EMS) and 15 nm (F’Ab) goat anti-rabbit (EMS) were applied at dilutions of 1/20 each, followed by fixation in 2.5% glutaraldehyde in dH_2_O. Grids were contrast-stained using a 2% aqueous uranyl acetate solution for 5 min and Reynold’s lead citrate (67) for 1 min.

Images were acquired at 120 kV on an FEI Tecnai G2 Spirit with an FEI Ceta 16 M camera (4k × 4k) (Thermo Fisher Scientific). Gold beads within a cytoplasmic protein aggregate were detected using the program imodfindbeads from IMOD analysis package (68), the resulting models corrected by hand and exported as wimp file. Nearest-neighbor analysis was performed by using the smallest value of distances between gold beads, calculated using Euclidian distance

$$\left(d=\sqrt[{2}]{{\left(x_{2}-x_{1}\right)}^{2}+{\left(y_{2}-y_{1}\right)}^{2}}\right)$$

The mean distances between nearest neighbors of GFP-labeling gold beads (15 nm) were compared with the mean distance between an RFP-labeling gold bead (25 nm) and the nearest GFP-labeling gold bead. The corresponding reverse analysis was performed, measuring between RFP-labeling gold beads and GFP-labeling gold beads to the nearest RFP-labeling gold beads. The numbers of beads analyzed to calculate the distance to each nearest neighboring bead were as follows: guk1-7-GFP gus1-3-mCherry set 15 nm to 15 nm 37 gold beads, 15 nm to 25 nm 12 gold beads, 25 nm to 25 nm 14 gold beads, 25 nm to 15 nm 28 gold beads; pro3-1-GFP gus1-3-mCherry set 15 nm to 15 nm 25 gold beads, 15 nm to 25 nm 7 gold beads, 25 nm to 25 nm 10 gold beads, 25 nm to 15 nm 15 gold beads. No statistically significant difference was found among the groups (Kruskal-Wallis rank sum test, *p*-value adjustment according to Holm, individual *p*-values shown in Table S2).

### Growth assays: drop test and liquid growth rates

To monitor fitness of all reporters at different temperatures, the strains were grown overnight in YPD, rediluted to A_600_ = 0.1, grown to mid-exponential phase, and all adjusted to A_600_ = 0.5. The cells were then serially diluted in 1/10 dilutions and spotted on YPD plates. The plates were incubated at 22, 30, 37, and 39 °C for two days and photographed. When indicated, azetidine-2-carboxylic acid was added to YPD agar at 0.6 mg/ml. Liquid growth rate assays were performed in multi-well plates in a BioTek Synergy 2 plate reader. Doubling time was determined from growth curves composed of OD readings measured every 60 min during exponential growth at 22, 30, and 38 °C starting at A_600_ = 0.01.

### Gel electrophoresis and immunoblotting

Cells were grown until mid-exponential phase (A_600_∼0.5) at 30 °C and shifted to 38 °C for heat shock experiments. Where indicated, cycloheximide was added to a final concentration of 250 μg/ml. At indicated time points, cells were harvested as 1 A_600_ units. Cells were resuspended in 200 μl of 0.2 M NaOH supplemented with 1 mM DTT, Complete Protease Inhibitor (Roche), and 2 mM Pefablock, incubated for 20 min on ice, pelleted, resuspended in 50 μl sample buffer (1× Laemmli buffer (80 mM Tris–HCl pH 6.8, 10 mM EDTA pH 8.0, 8% SDS, 20% glycerol, 0.004% bromophenol blue), 8 M Urea, 2.5% β-mercaptoethanol) and heated for 10 min at 70 °C. Fifteen microliters supernatant was typically loaded per lane on a 4 to 12% gradient 26 well Criterion XT Bis-Tris Protein gel (Bio-Rad). Gels were transferred on PVDF membranes with a wet blotting system (Criterion blotter, Bio-Rad). Membranes were probed with anti-GFP (ab6556; Abcam, Cambridge, UK, 1/20,000 dilution), anti-Rpn5 when indicated (ab79773; Abcam, Cambridge, UK, 1/20,000 dilution), and anti-Pgk1 (ab90787; Abcam, Cambridge, UK, 1/20,000 dilution) as a loading control. As secondary antibodies, goat anti-mouse IRDye 800CW and goat anti-rabbit IRDye 680 or goat anti-rabbit IRDye 800CW and goat anti-mouse IRDye 680LT (LI-COR; 1/20,000 dilution) were used. Membranes were scanned using the LI-COR Odyssey Infrared scanner. Images were quantified with Fiji software.

### MG132 treatment for degradation assay

Cells were grown to early exponential phase (A_600_∼0.3–0.35) at 30 °C. SDS was added to the cultures destined for DMSO control and MG132 treatment at 0.003% for 90 min. DMSO or MG132 (75 μM) was added to the cultures for 30 min. Lastly, cycloheximide was added to a concentration of 250 μg/ml directly before shifting the cells to 38 °C for heat shock (time point 0 min).

### GFP-pulldown for detection of ubiquitylation

All steps after cell growth were performed at 4 °C or on ice. Cells were grown to mid-exponential phase (A_600_∼0.5) at 30 °C and harvested as 50 A_600_ units, washed twice with H_2_O, and once with IP buffer B (115 mM KCl, 5 mM NaCl, 2 mM MgCl_2_, 1 mM EDTA pH 8.0, 20 mM Hepes/KOH pH 7.5). The cell pellets were resolved in 250 μl IP buffer B supplemented with inhibitors (1 mM DTT, Complete Protease Inhibitor (Roche), 2 mM Pefablock, and 20 mM NEM). Cells were lysed using 150 μl glass beads and 10 cycles of vortexing and chilling. Lysates were incubated with 0.5% Nonidet-P40 (Roche) for 20 min and centrifuged (8000 rpm for 20 min). Precleared lysates were incubated with 20 μl bed volume of washed GFP-TrapA beads (Chromotek) for 1 hour in a rotator, centrifuged (2000 rpm), and washed four times with IP buffer B with 0.5% Nonidet-P40. The beads were then boiled at 95 °C for 5 min in 50 μl 2× Laemmli buffer with 5% β-mercaptoethanol and subjected to gel electrophoresis and immunoblotting as described above. The antibody used to detect ubiquitin was anti-Ubiquitin (ab19247; Abcam, Cambridge, UK, 1/1000).

### Replicative lifespan analysis

Replicative lifespan was assessed as previously described (69) using the MSM 400 (Singer Instruments) micromanipulator. Briefly, cells were grown overnight in YPD to early logarithmic phase (A_600_ = 0.1–0.3), plated, and manually dissected until they did not produce any daughters. Each replicative lifespan analysis was performed independently three times.

### Statistical analysis

Data from quantifications are presented as the average or median of ≥3 biological experiments ± SEM unless indicated otherwise. Aggregate quantifications of cells are based on counting ≥200 cells per strain, condition, and replicate. Aggregate lifespan was determined for 20 colocalized and 20 pro3-1-GFP aggregates each in three biological replicates. Data analysis and visualization were performed with GraphPad Prism 8.3.1. In figures, asterisks denote statistical significance determined by the indicated statistical test with ns *p* > 0.05, ∗*p* ≤ 0.05, ∗∗*p* ≤ 0.01, ∗∗∗*p* ≤ 0.001, ∗∗∗∗*p* < 0.0001.

## Data availability

All data are contained within this article.

## Supporting information

This article contains supporting information (17, 53, 70, 71).

## Conflict of interest statement

A. M. E.-B. and F. E. are now employed by AstraZeneca, Gothenburg, Sweden; L. L. B. is employed by Cochlear bone anchored solutions, Mölnlycke, Sweden. The authors declare that they have no conflicts of interest with the contents of this article.
