# Supplementary material for: Using reporters of different misfolded proteins reveals differential strategies in processing protein aggregates
Source: J Biol Chem. 2022 Sep 9;298(11):102476. doi: 10.1016/j.jbc.2022.102476 (PMC9636550; doi:10.1016/j.jbc.2022.102476)
Supplement: Supplemental Figures S1–S6 [file mmc1.pdf]

Supporting information

Using reporters of different misfolded proteins reveals differential strategies in processing protein aggregates

Kara L. Schneider, Doryaneh Ahmadpour, Katharina S. Keuenhof, Anna Maria Eisele-Bürger, Lisa Larsson Berglund, Frederik Eisele, Roja Babazadeh, Johanna L. Höög, Thomas Nyström and Per O. Widlund

The supporting information contains:

Figures S1-S6

Table S1 (Excel file)

Table S2 (Excel file)

Figure S1

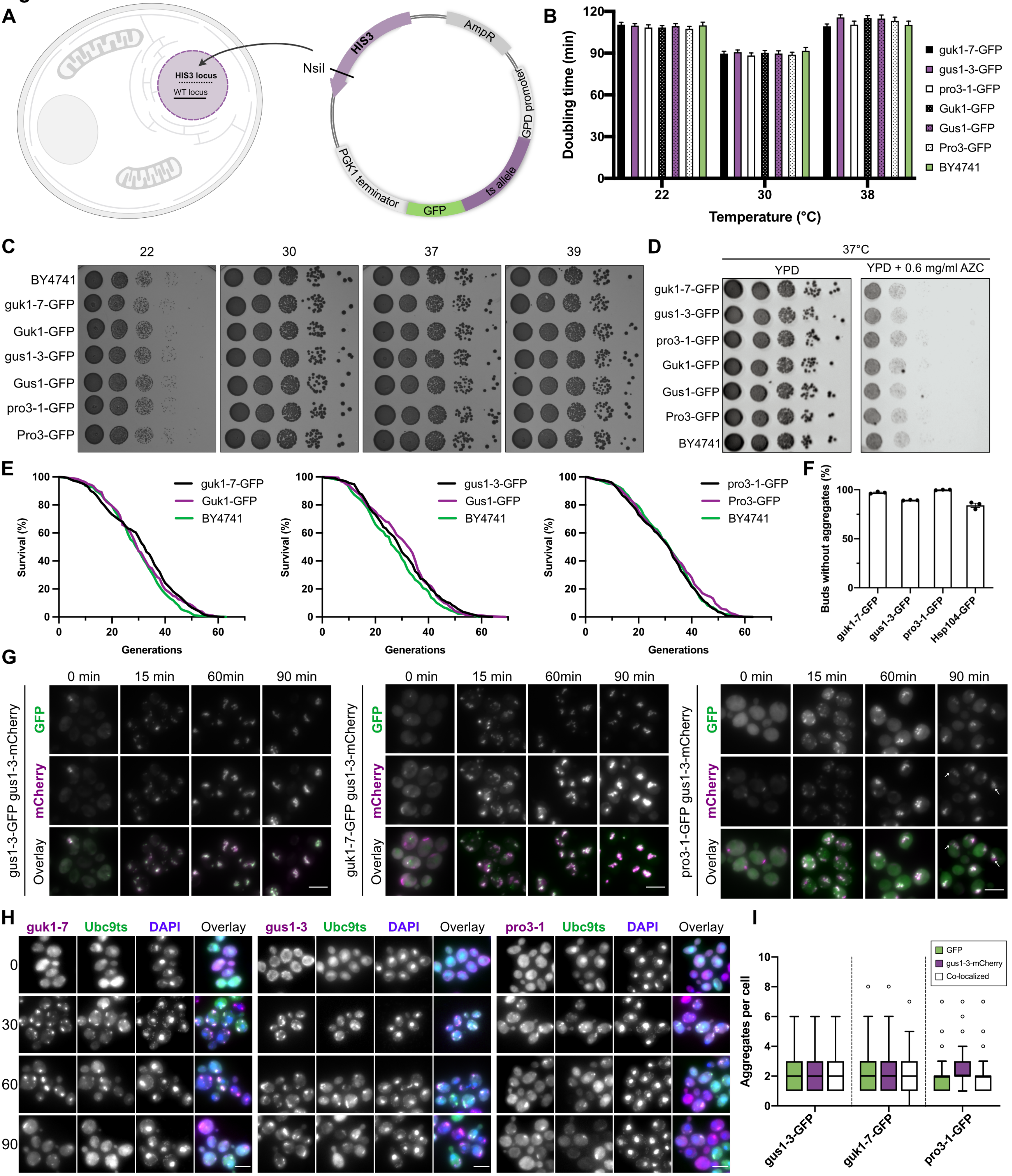

Supplementary figure 1: The misfolding reporter proteins are not toxic to the cell. (A) Scheme of plasmid and integration strategy of the ts alleles. (B) Doubling time during logarithmic growth of the ts and WT alleles and

BY4741 at 22, 30 and 38°C. (C) Spot test of the ts and WT alleles and BY4741. Cells were grown to OD_{600}=0.5, spotted on YPD and incubated at 22, 30, 37 and 39°C for 3 days. (D) Spot test of the ts and WT with AZC treatment. Cells were grown to OD_{600}=0.5, spotted on YPD and YPD+AZC and incubated at 37°C. (E) Replicative lifespan analyses of the ts and WT alleles and BY4741. (F) Asymmetry analysis of misfolding reporter and endogenous protein aggregates after heat shock at 38°C for 90 min. (G) Time course of strains containing a pairwise combination of reporters at 38°C. Grayscale images of GFP and mCherry levels are not adjusted, images labeled Overlay are adjusted to better visualize co-localization. Arrows indicate gus1-3-mCherry aggregates that do not co-localize with pro3-1-GFP at time point 90 min. (H) Time course of strains containing a pairwise combination of each mCherry-tagged misfolding reporter and Ubc9ts-GFP. Numbers indicate minutes at 38°C. DAPI stain visualizes DNA. (I) Quantification of cells in (G) presented as a box plot (Tukey method). Scale bar, 5 µm.

Figure S2

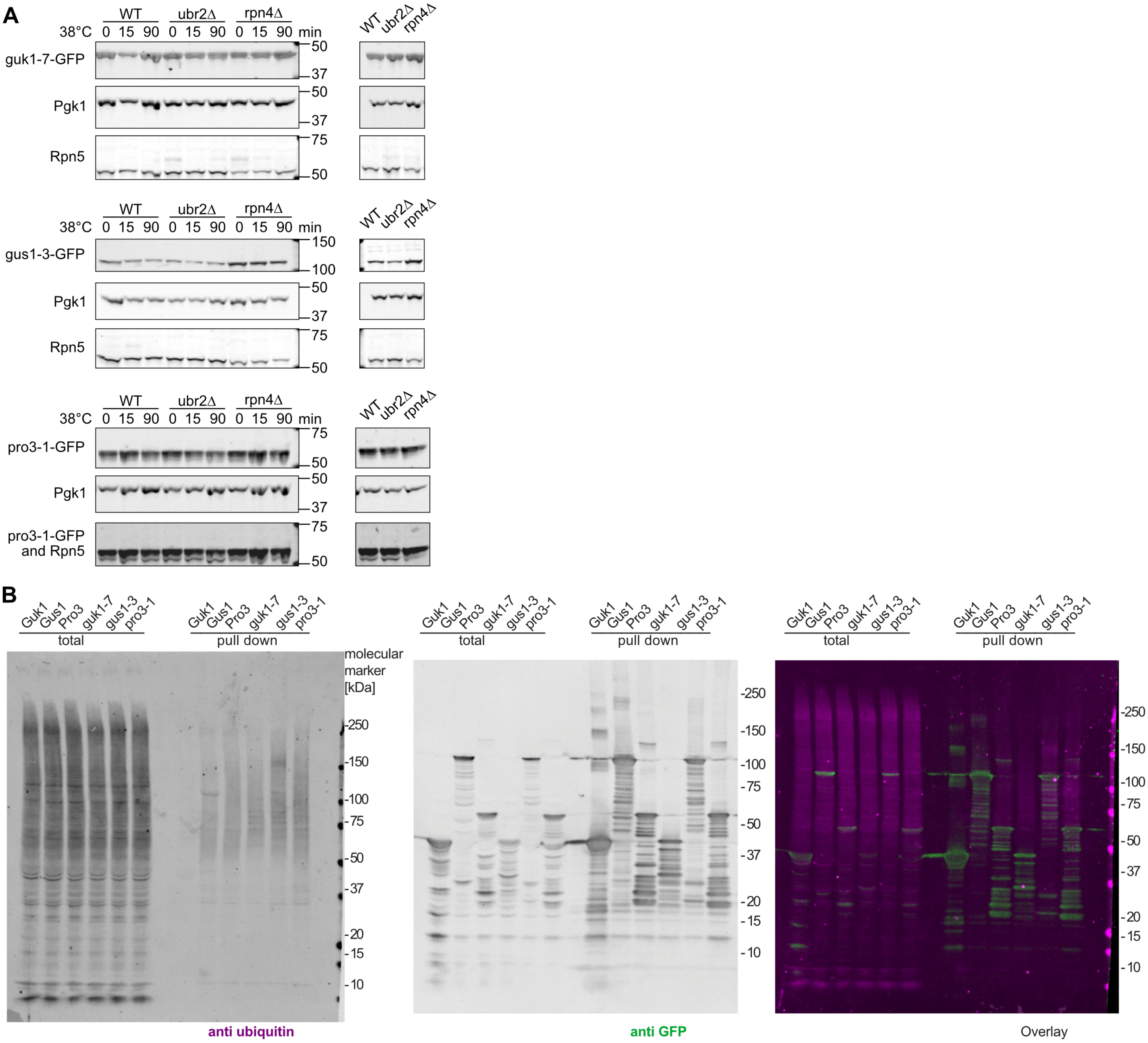

Supplementary figure 2: The misfolding reporters and Gus1-GFP and Pro3-GFP are ubiquitylated. (A) Representative blot of cells in Figure 2D. Immunodetection was performed against the ts proteins, Rpn5 (proteasome subunit) and Pgk1 (loading control). Note increased Rpn5 levels in *ubr2*Δ and decreased levels in *rpn4*Δ cells throughout heat shock at 38°C (left) and at 30°C (right). (B) Ubiquitylation assay of GFP-pulldown of ts and WT reporters shows increased ubiquitylation of the three ts reporters at 30°C in comparison to the corresponding wild type.

Figure S3

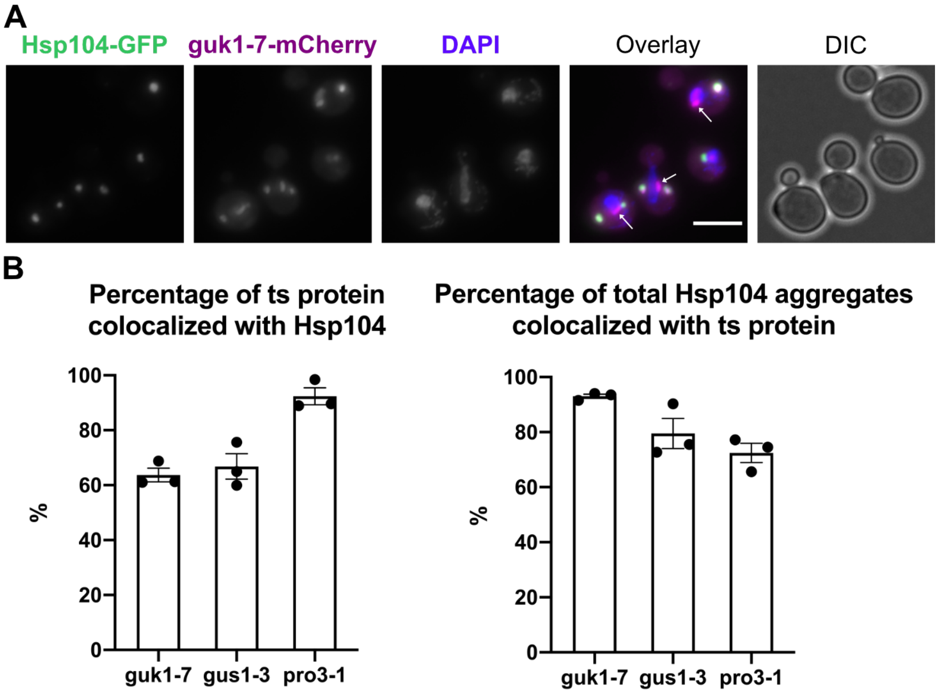

Supplementary figure 3: The misfolding reporters co-localize with Hsp104-GFP. (A) The "Hsp104-free" aggregate of guk1-7-mCherry localizes to the nucleus to a large extent. Cells from Figure 3A were stained with DAPI. Overlay images are enhanced in brightness and contrast to increase visibility. Arrows indicate "Hsp104-free" aggregates of guk1-7-mCherry at the nucleus. (B) Alternate visualization of data in Figure 3B. Almost all for co-localization available pro3-1-mCherry aggregates co-localize with Hsp104 and almost all available Hsp104-GFP aggregates co-localize with guk1-7-mCherry. Scale bar, 5 µm.

Figure S4

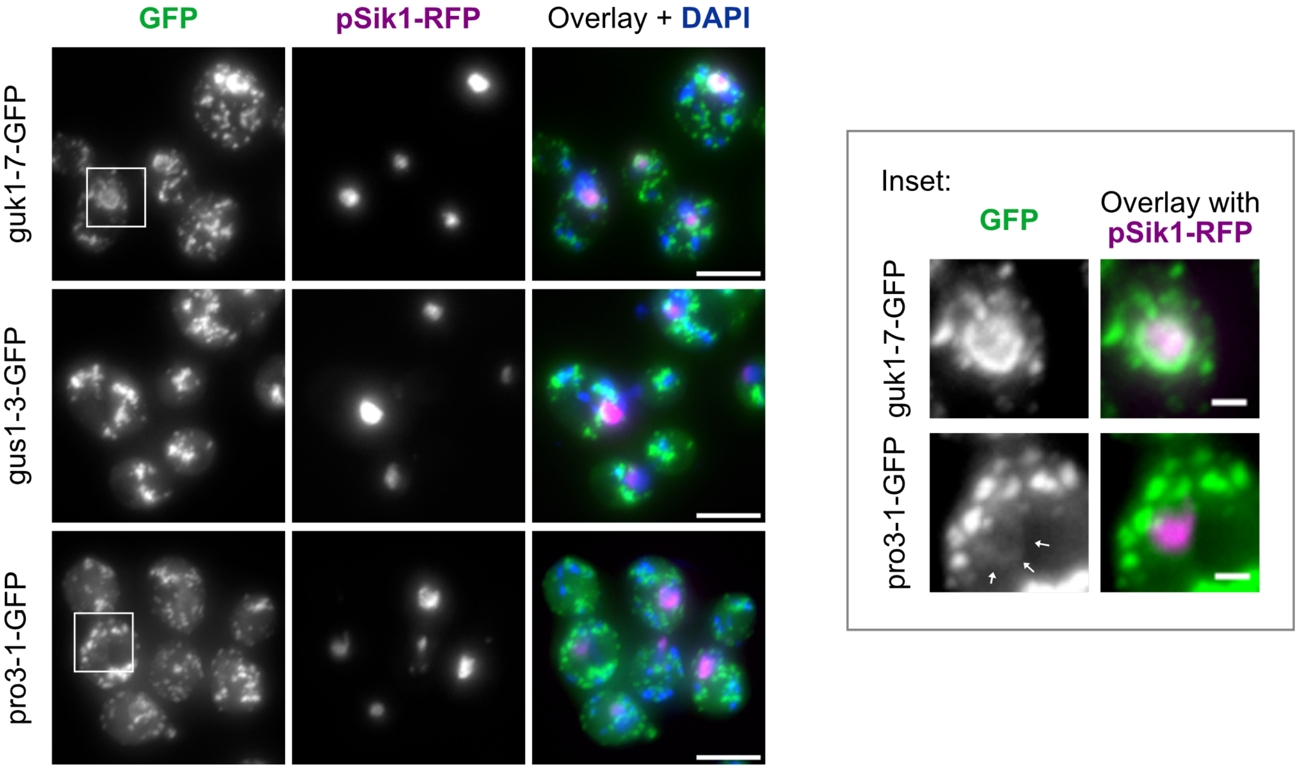

Supplementary figure 4: The misfolding reporters guk1-7-GFP and pro3-1-GFP form aggregates around the nucleolus. Live cells imaged after 30 min heat shock at 42°C and subsequent live-cell DAPI staining. Images are adjusted in brightness and contrast (for pro3-1-GFP also gamma adjustments) to increase visibility of nucleolar aggregate structure. White rectangles indicate location of inset. Arrows point to the nucleolar pro3-1-GFP structure. Scale bar, 5 µm (Inset, 1 µm).

Figure S5

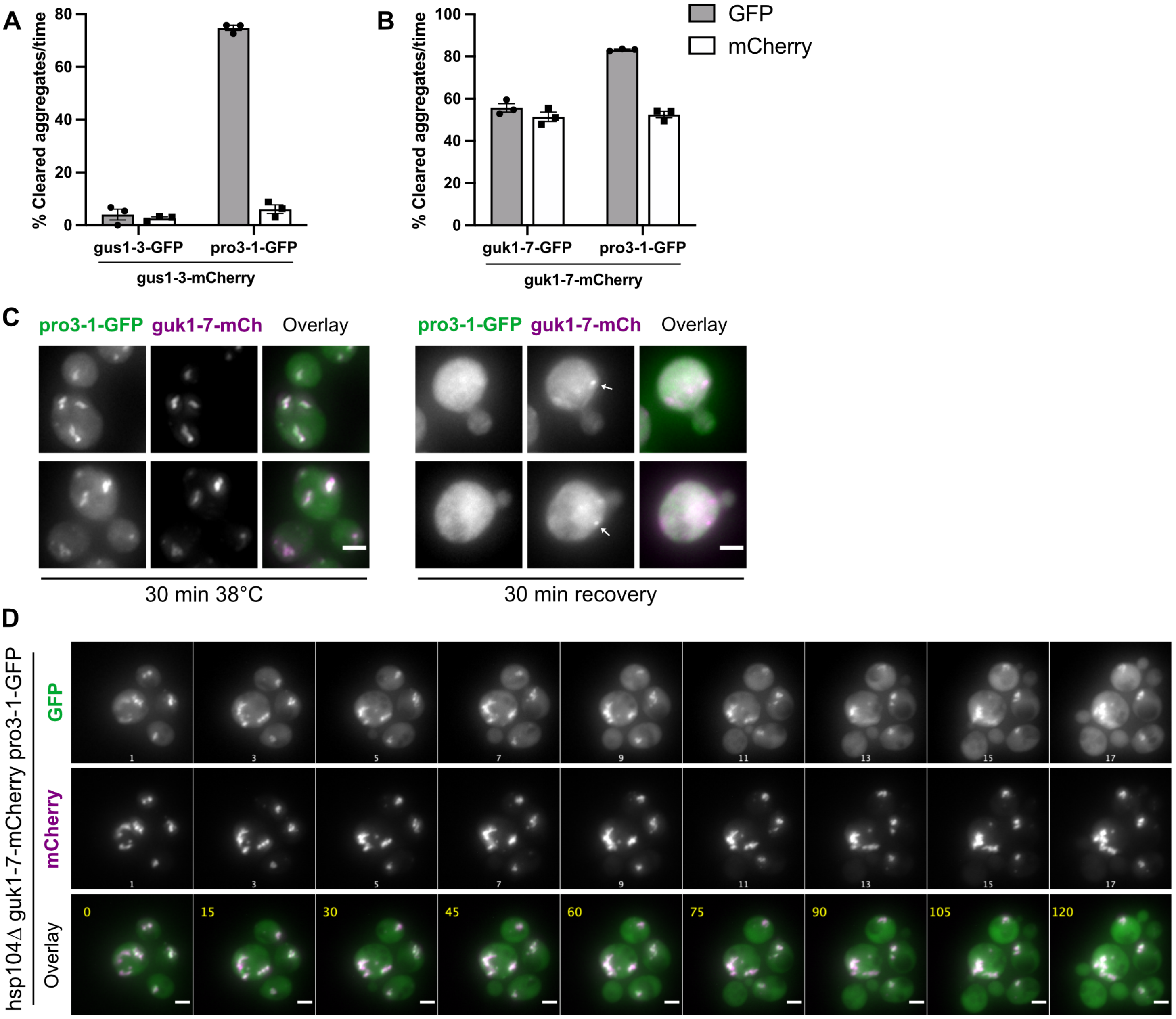

Supplementary figure 5: pro3-1-GFP clearance is not affected in combination with gus1-3- or guk1-7-mCherry. (A) gus1-3-mCherry was combined with gus1-3-GFP as a control and with pro3-1-GFP and clearance of aggregates over time was monitored after heat shock at 38°C for 60 minutes and recovery at 30°C for 60 minutes. (B) guk1-7-mCherry was combined with guk1-7-GFP and with pro3-1-GFP and clearance of aggregates over time was monitored after heat shock at 38°C for 30 minutes and recovery at 30°C for 30 minutes. (C) Representative cells of the quantification in (B). Arrows indicate examples of visible guk1-7-mCherry aggregates in absence of pro3-1-GFP aggregates. (D) Time lapse microscopy montage of pro3-1-GFP guk1-7-mCherry cells in *hsp104Δ* background during recovery at 30℃ after heat shock at 38℃ for 60 minutes. Time stamp in min (yellow), montage increment=2 (white), Scale bar, 2 µm.

Figure S6

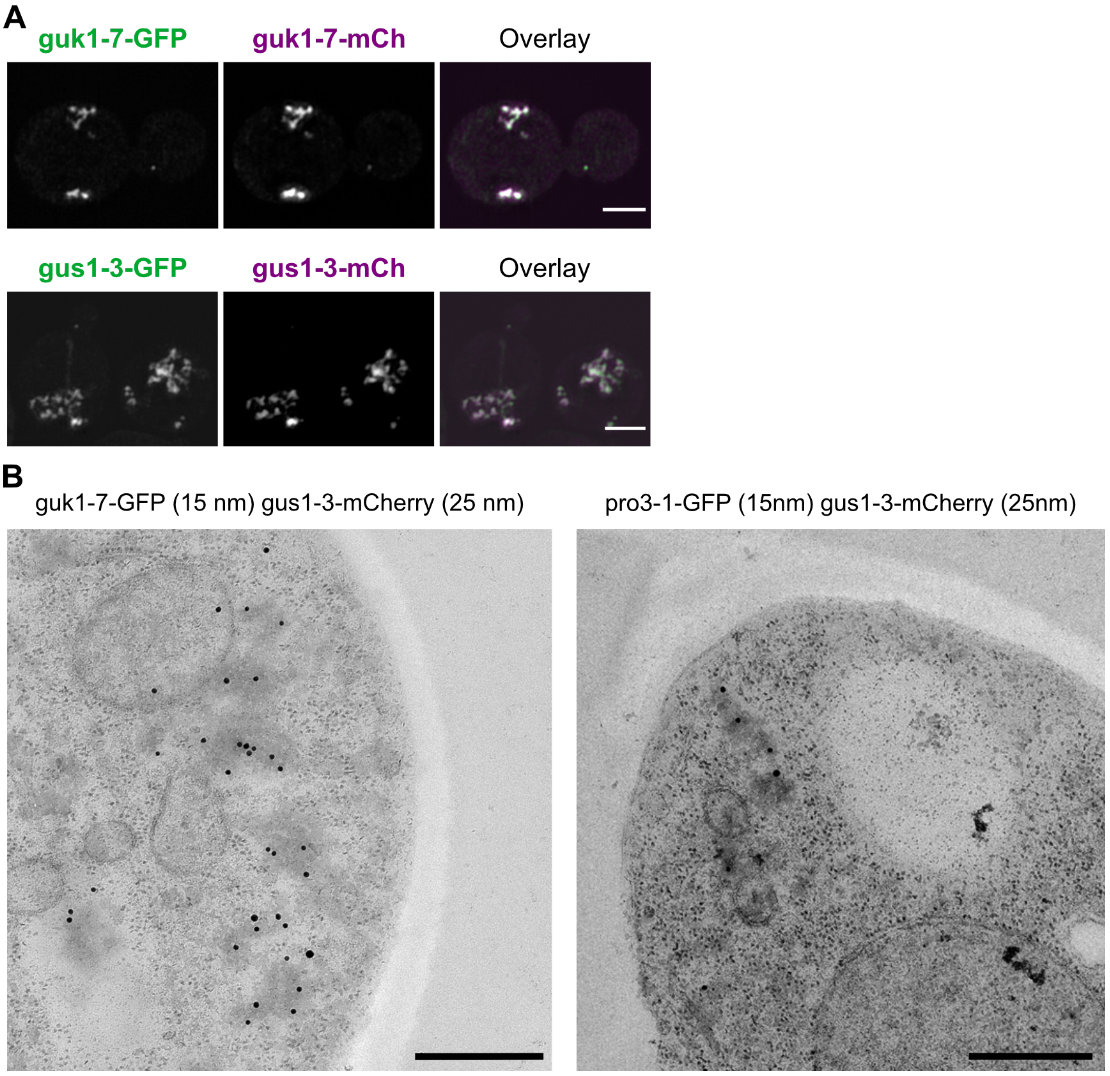

Supplementary figure 6: The different protein species are mixed within common inclusions. (A) 3D-SIM images as maximum Z-projections of aggregates of the control strains guk1-7-GFP guk1-7-mCherry and gus1-3-GFP gus1-3-mCherry after 60 min at 38°C. Scale bar, 2 µm. (B) Representative EM images of double immunogold labeling of high pressure-frozen yeast cells of guk1-7-GFP gus1-3-mCherry (18500x magnification, left) and pro3-1-GFP gus1-3-mCherry (11000x magnification, right) after heat shock at 38°C for 30 min. 15 nm gold beads were attached to the anti-GFP antibody, 25 nm gold beads to anti-RFP. Scale bar, 500 nm.
